# Recent Advances in Electrochemical Biosensors: Applications, Challenges, and Future Scope

**DOI:** 10.3390/bios11090336

**Published:** 2021-09-14

**Authors:** Anoop Singh, Asha Sharma, Aamir Ahmed, Ashok K. Sundramoorthy, Hidemitsu Furukawa, Sandeep Arya, Ajit Khosla

**Affiliations:** 1 Department of Physics, University of Jammu, Jammu 180006, India; anoopsinghchib1111@gmail.com (A.S.); psashasharma941@gmail.com (A.S.); ahmedaam131@gmail.com (A.A.); 2 Department of Chemistry, SRM Institute of Science and Technology, Kattankulathur 603203, India; ashokkus@srmist.edu.in; 3 Department of Mechanical System Engineering, Graduate School of Science and Engineering, Yamagata University, Yamagata 992-8510, Japan; furukawa@yz.yamagata-u.ac.jp

**Keywords:** biosensor, electrochemical, sensitivity, amperometric, voltammetric, food quality monitoring, machine learning

## Abstract

The electrochemical biosensors are a class of biosensors which convert biological information such as analyte concentration that is a biological recognition element (biochemical receptor) into current or voltage. Electrochemical biosensors depict propitious diagnostic technology which can detect biomarkers in body fluids such as sweat, blood, feces, or urine. Combinations of suitable immobilization techniques with effective transducers give rise to an efficient biosensor. They have been employed in the food industry, medical sciences, defense, studying plant biology, etc. While sensing complex structures and entities, a large data is obtained, and it becomes difficult to manually interpret all the data. Machine learning helps in interpreting large sensing data. In the case of biosensors, the presence of impurity affects the performance of the sensor and machine learning helps in removing signals obtained from the contaminants to obtain a high sensitivity. In this review, we discuss different types of biosensors along with their applications and the benefits of machine learning. This is followed by a discussion on the challenges, missing gaps in the knowledge, and solutions in the field of electrochemical biosensors. This review aims to serve as a valuable resource for scientists and engineers entering the interdisciplinary field of electrochemical biosensors. Furthermore, this review provides insight into the type of electrochemical biosensors, their applications, the importance of machine learning (ML) in biosensing, and challenges and future outlook.

## 1. Introduction

An analytical device that provides information about a biological process through a transducer is called a biosensor. The information can both be semi-measurable and measurable. The biochemical receptor is used to collect information about the biological process and then transferred to the transducer. An ideal biosensor should be temperature and pH-independent, recyclable, and specific. It was Cammann who first invented the term “biosensor” and the definition of the biosensor was introduced by IUPAC [1]. Biosensor fabrication is a multidisciplinary field as it involves chemistry, biology, physics, electronics, material science, engineering, etc. [2]. Recently, various new elements, such as molecularly imprinted polymers, aptamers, nanomaterials, etc., have been developed in the field of biosensors [3,4,5,6,7]. The growth of nanotechnology has provided strong benefits to the biosensor field as the nanomaterials have a large surface-to-volume ratio that helps in achieving higher sensitivity and efficiency [8]. In most of the biosensors developed so far, electrochemical detection has been used in one way or another, i.e., amperometric, voltammetric, and impedimetric detection [9]. Moreover, the catalytic biosensor that uses some kind of enzymes covers half of the production of biosensors, whereas the affinity biosensors using antibodies represent the other half [10]. The affinity biosensors are mostly used in monitoring the quality and safety of food products along with DNA (de-oxyribose nucleic acid) detection in reactions (hybridization) [11]. The electrochemical biosensors consist of three electrodes, i.e., reference, counter, and working. The electron generated or consumed during the catalysis of enzymes is used for producing a signal that helps in the detection process [12]. These are further classified as potentiometric, amperometric, voltammetric, and impedimetric biosensors. Recently, computers have made a walk-in in every field of science and technology. Machine learning is an important aspect of the computer language program and has been used in monitoring and refining various data outputs. While sensing complex structures and entities, large data are obtained, and it becomes difficult to manually interpret all the data. Machine learning helps in interpreting large sensing data and has been used in various applications. Moreover, the data obtained sometimes are not clear and possess some noise and disturbance. Machine learning can help in analyzing such data and obtain the required results. In the case of biosensors, the presence of impurity affects the performance of the sensor and machine learning helps in removing the signals obtained from the contaminants to obtain a high sensitivity. Moreover, the interpretation of the data becomes quite easy and effective by using machine learning in the biosensing process [13,14,15]. In this review, various machine learning tools and techniques that are used for biosensing are discussed briefly along with their applications and limitations.

## 2. Types of Biosensors

### 2.1. Catalytic Biosensors

**Figure 1:**
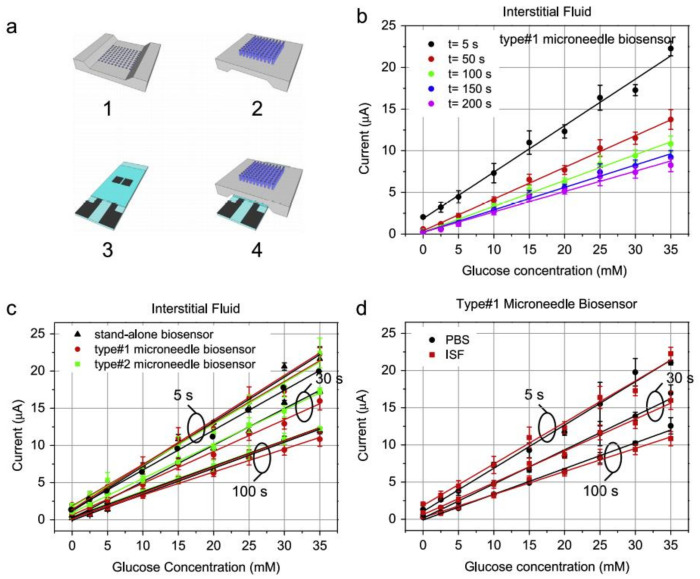
(**a**) Representation of the biosensor having glucose sensor attached on the back of the microneedle; (**b**) Calibration curves for the concentration between 0–35 mM; (**c**) Calibration curves of developed biosensors and stand-alone biosensors in ISF, and (**d**) Calibration curves for developed biosensor for concentration 0–35 mM at 5 s, 30 s, and 100 s of sampling times. Reproduced from Reference [20] with permission. Copyright 2015, Elsevier.

The development of classical first-generation enzyme electrodes has started some five decades ago. The procedure involves depositing enzyme on the surface of the transducer and the signal produced depends upon the products produced on the transducer surface after the reaction and diffusion of the target on the substrate. To improve efficiency and lessen the enzyme electrode’s cost, different types of electrodes are specifically modified using nanomaterials and sometimes replaced by carbon electrodes. Cinti et al. [16] developed a cholesterol biosensor using screen-printed electrodes that were modified using nanoparticles of Prussian blue. The hydrogen peroxide produced due to the oxidase enzyme can be detected with such a setup. Sannini et al. also explained this by developing a lactate biosensor that can be used to monitor fermentation in an interferent-rich matrix-like wine [17]. There has been the detection of spermine and spermidine, which are polyamines, which can help in monitoring food safety and human health. This method employs newly designed oxidase, i.e., spermine and polyamine. The oxidase is captured in the gel of polyvinyl alcohol, and carbon-based electrodes that are modified with Prussian blue are used for carrying out the reduction process [18]. Another method is to create a barrier on the electrode surface and entrapping the enzyme on the layer of the electrode surface by physical entrapment or cross-linking. For a selective membrane, an ideal contender is over-oxidized polypyrrole. A Pt electrode having lysine oxidase entrapped on it is also used as a biosensor for detecting maturation [19]. The biosensors based on glucose oxidase have been mostly used for glucose sensing. Strambini et al. developed a microneedle-shaped biosensor that can monitor glycaemia present in the intestinal fluids [20]. They mounted a glucose biosensor on the back of the needle that can detect glucose present in the intestinal fluid with an accuracy of ±20% only in 30 s. The biosensor worked on the capillary action and can be used for real samples [21]. Figure 1a shows the design of the biosensor where the glucose sensor is mounted on the back of a needle-shaped chip. The glucose sensor comprises graphite electrodes having glucose oxidase entrapped into the pores. Figure 1b shows the calibration curves and Figure 1c,d shows the experimental data depicting that the sensor can be used to detect concentrations as low as 0 to 35 mM with a sampling time of 5, 30, and 100 s.

**Figure 2:**
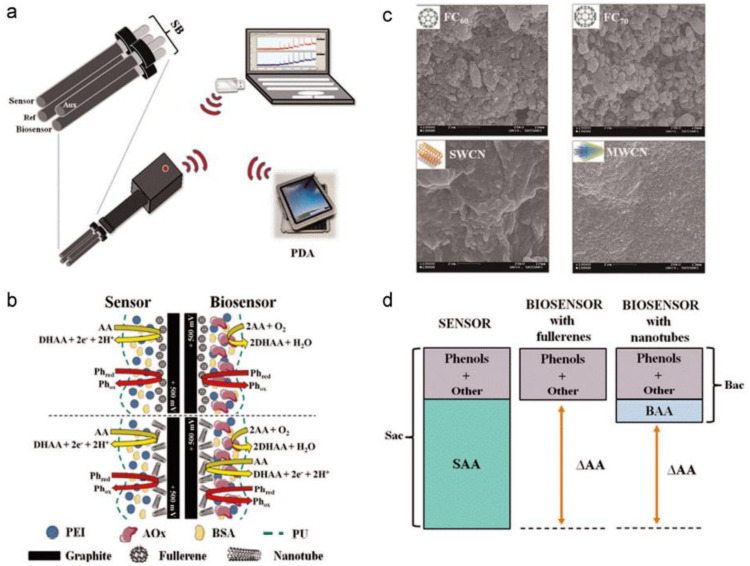
(**a**) Pseudoreference, working, and auxiliary electrodes; (**b**) CSEM images of biosensor surface modified with different materials. (**c**) Diagrammatic representation of the sensors with the modified surface; (**d**) working of the sensor. Reproduced from Reference [24] with permission. Copyright 2015, Elsevier.

The second-generation enzyme electrodes that have been developed by depositing gold on PC membranes have also been used in manufacturing electrochemical biosensors. By using the mediator as (Ferrocenylmethyl)trimethylammonium, the limit of detection (LOD) of the glucose was 36 µM. The fouling of the electrode surface can also be recognized due to oxidation which does not exist in the first-generation electrodes. Thus, first-generation electrodes can be used to prevent their fouling. Malvano et al. [22] used alcohol dehydrogenase and entrapped it into an SPE electrode customized with poly(2-acrylamido-2-methyl-1-propane) sulfonic acid polymer doped in polyaniline which helped in preventing the fouling. Functional parameters can also be calculated using enzyme electrodes, as the food contains antioxidants (produced by chemicals in the food). The enzyme-based biosensors have been used for detecting the antioxidants produced from yogurt and berry juices [23]. Moreover, these biosensors have also been used to monitor antioxidants and ascorbic acid in orange juice, blueberry, and kiwi using modified graphite and fullerenes as shown in Figure 2a [24]. In Figure 2b, the CSEM (Cryo scanning electron microscopic) images of the biosensor are shown, whereas Figure 2c,d represents the schematic diagram and working of the biosensor, respectively. Due to the oxidation of the enzyme, there was a decrease in the current which helps in monitoring and measuring the ascorbate present in the sample.

It must be noted that enzyme-based electrodes have been used for sensing pesticides, but with advancement and technology, they can be used for monitoring food safety. Atrazine is an important component of pesticides. The activity of the enzyme tyrosinase can be inhibited by the atrazine chemical. The electrode-based on tyrosinase has been used for detecting atrazine with 0.3 ppm LOD in the water bodies [25]. Similarly, an enzyme-based electrode of bilirubin oxidase has been used to monitor the oxygen level of a microbial fuel cell having a 0–0.08 mW power range [26]. The enzymes such as urease, d-lactate dehydrogenase, acetylcholinesterase, and β-galactosidase have been used in catalytic biosensors for optical detection of milk quality and safety [27].

### 2.2. Affinity Biosensors

**Figure 3:**
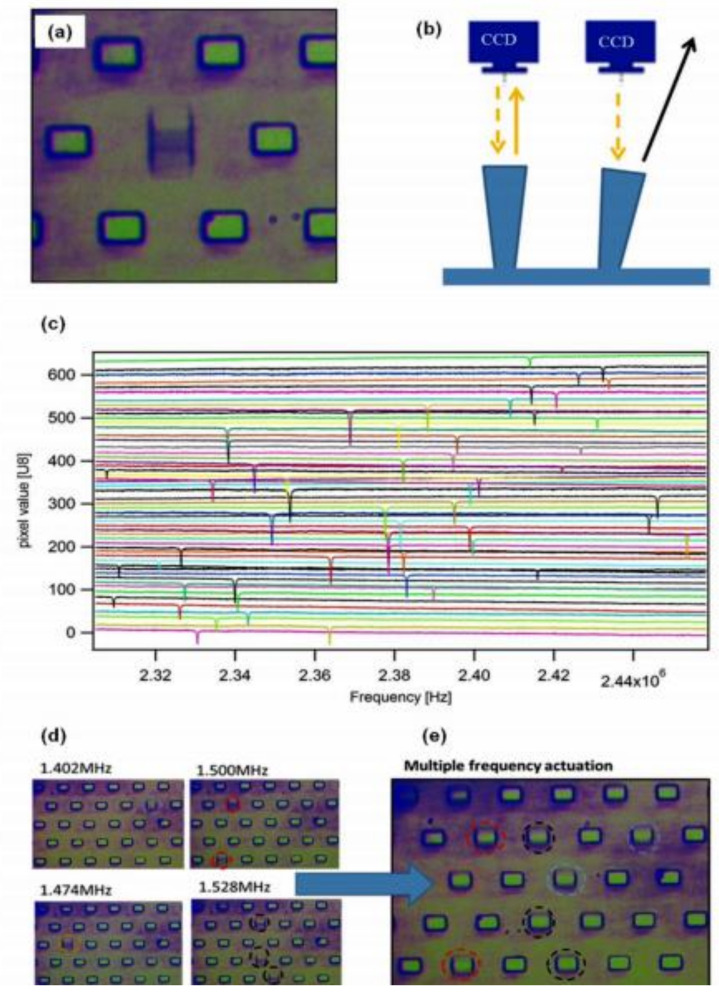
(**a**) T-shaped pillar array and its optical image; (**b**) Process of measuring the intensity as a function of frequency; (**c**) 53 traces corresponding to 53 different pillars as a function of actuation frequency. Actuation mode, (**d**) single-mode, and (**e**) multiple modes. Reproduced from Reference [29] with permission. Copyright 2015, Elsevier.

The term affinity biosensor in this article refers to a device incorporating immobilized biological receptor molecules that can reversibly detect receptor–ligand interactions with a high differential selectivity and in a non-destructive fashion. In affinity biosensors, antibodies are used as biochemical agents for detecting the biological process. Immunosensor is an important class of affinity biosensors. In the last four decades, the focus in the development of immunosensors has been reducing incubation time, better design, signal amplification, label-free detection, and control over antibody placement. When it comes to the sensitivity and production cost of such immunosensors, the enzyme-linked immunosorbent assay (ELISA) technique has been used most widely. A label-free immunosensor based on optical fiber was fabricated using a thin film of titania–silica dip-coated over the sensing area of the fiber [28]. The analyte sensing was done by measuring the wavelength at the initial and endpoint of the fibre. The fabricated immunosensor was successfully used for detecting Immunoglobin (IgG) and anti-IgG in human serum with very low LOD. In another example, Tardivo et al. [29] reported a sensor having thousands of micropillars and columnar resonators arranged in an area of 1 mm square. The sensing was done using image and software analysis capturing images at 15 fps. Since there are various sensors incorporated, thus the variation obtained in the frequency measurement by a single sensor is neglected. Figure 3a,b shows an optical image and the process of measuring the intensity of the image as a function of frequency, whereas Figure 3c represents 53 traces obtained for 53 pillars, and Figure 3d,e shows the tracking of the parallel pillars at a resonant frequency in real-time.

**Figure 4:**
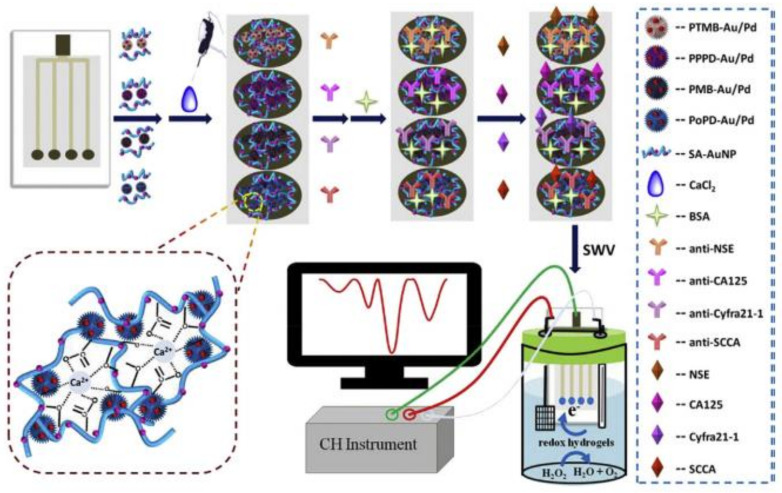
Multiplexed label-free amperometric immunosensor based on SPCE modified by Au-pd NPs and some polymeric matrices for the detection of some biomarkers. Reproduced from Reference [31] with permission. Copyright 2018, Elsevier.

For detecting a prostate antigen called interleukin, which is an inflammatory cytokine, an immunosensor with a detection range from 0.3 to 100 nM was fabricated using an electrolyte-gated organic field-effect transistor (EGOFET) [30]. Moreover, ELISA which is a complete and simple detection format, can be easily used with mobile phones, with mobile phones acting as a detector. In Figure 4, Zhao et al. proposed an improved screen-printed carbon electrode (SPCE) for multiplexed label-free amperometric immunosensor. It is observed that the limit of detection was 5.5 pg mL^−1^ and the sensitivity of this immunosensor was 0.83 µA (lg(ng ml^−1^))^−1^ [31].

**Figure 5:**
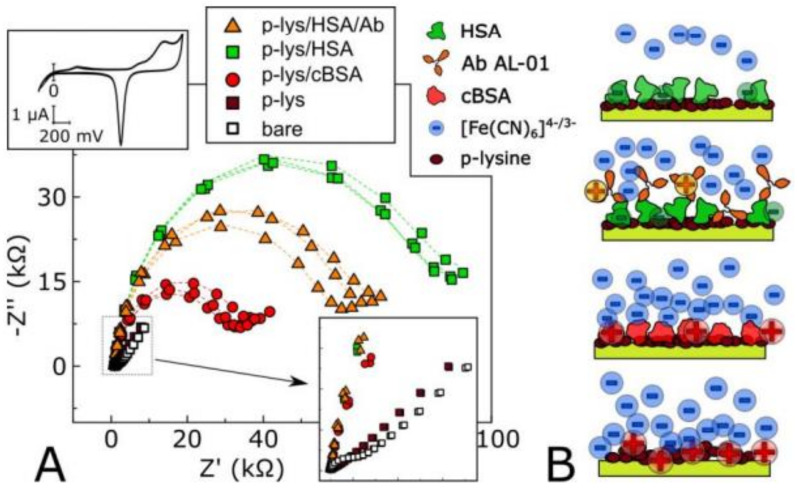
(**A**) Schematic visualization of the processes on the electrode. (**B**) Corresponding gel electrophoresis proving the charge of utilized compounds upon experimental pH. Reproduced from Reference [42] with permission. Copyright 2018, Elsevier.

Aptamers can also be used for detecting antibodies. The aptamers are ribonucleic acid (RNA) and DNA molecules (single-stranded) that are highly selective and bind to particular antibodies, thus helping in their efficient detection. Different aptamers have different affinities based on which they can be used in developing biosensors with low LOD value. Castillo et al. [32] used a dendrimer structure for developing a voltammetric biosensor for sensing a mycotoxin called AFB1 (aflatoxin B1) in food products. The biosensor had a LOD of 0.40 nM for concentrations of 0.1–10 nM of AFB1. For the case study, the biosensor was tested on polluted peanut extract and peanut snacks. Scarano et al. [33] fabricated a piezoelectric material-based biosensor to detect this protein in human blood serum. Tintore et al. [34] used two aptamers with gold-coated magnetic nanoparticles for detecting single methylation in the DNA of aptamer using α-thrombin protein and plasmonic detection. The protein usually causes the agglomeration of nanoparticles resulting in the variation of color and UV of the solution. However, in this case, one of the aptamers prevented the agglomeration of the nanoparticles via single methylation of the DNA. Thus, the biosensor developed can be used to detect DNA modifications. Ravalli et al. [35] developed a biosensor based on an impedance study for detecting human epidermal growth factor receptor 2 (HER2). The antibody used for the detection of analyte was a protein produced using biotech engineering. The biosensor can detect a 0 to 40 µg/L concentration of HER2 with an LOD of about 6 µg/L. The albumin of the rat and human serum can bind with bicalutamide. In one such work, a transistor was developed where the gate of the transistor was modified with an odorant protein [36]. Pietrantonio et al. [37] deposited an odorant protein such as porcine, bovine, and double-mutant bovine protein on the surface of a wave resonator using the LIFT technique. The biosensor successfully distinguished between the carvone and octanol molecules that are present in the molds. Thus, the biosensor can be used for monitoring food products against molds. Cennamo et al. [38] detected butanal using an odorant protein called porcine. The biosensor selectively detected the butanal with concentrations ranging from 20–1000 µM. Compagnone et al. [39] developed a biosensor using gold-modified quartz crystal arrays. The biosensor was able to detect 95% of the off-flavored chocolate sample using the PLS-DA analysis technique. Zuccaro et al. [40] developed a graphene-based biosensor that can monitor the behavior of the enzyme topoisomerase IB present in the human DNA. The interaction between the enzyme and the graphene substrate was analyzed by determining the field-effect characteristics of the biosensor. The biosensor provided real-time analysis and can be used for screening drugs in the future. The hybridization of DNA is an important feature whose detection is very helpful in various fields of biology. The biosensors with high selectivity and sensitivity can be used for detecting DNA hybridization. Mariani et al. [41] reported a method for detecting genomic human DNA (obtained from lymphocytes) without using the polymerase chain reaction (PCR) amplification process. The nanostars of gold were arranged in a sandwich-type model depending upon the analyte to be detected in the DNA and surface Plasmon resonance (SPR) imaging tool was used as a detector. A LOD of 10 pM was obtained with this method, which is low as compared to other biosensors developed, i.e., 6.1 nM. In another published work, Karel Lacina et al. [42] observed decrease in the impedimetric response (as shown in Figure 5) due to the binding of positively charged proteins. These assumptions were confirmed using gel electrophoresis which seems promising as a simple tool for such applications.

Electrochemical methods can be used for sensing the hybridization of DNA. In one work reported, the electrochemical biosensor with an enzyme was used to detect the modified and unmodified DNA in PCR samples [43]. In another work, gold nanoparticles were incorporated as dots on a reduced graphene oxide electrode to produce an aptamer-based electrochemical biosensor [44].

## 3. Electrochemical Biosensors

The electrochemical biosensor is a special type of biosensor where a biological entity is detected by converting the information into an electrical signal, i.e., voltage, current, impedance, etc. [45,46]. The first electrochemical biosensor was developed by Clark to monitor the glucose level in human blood serum. The electrochemical biosensors have been developed to detect various biological entities such as enzymes, proteins, viruses, antibodies, etc. [47]. The electrode is the most vital component of an electrochemical biosensor as it controls the flow of electrons and bioagents. Various electrochemical biosensors that have been developed will be discussed in this section.

### 3.1. Amperometric Biosensor

**Figure 6:**
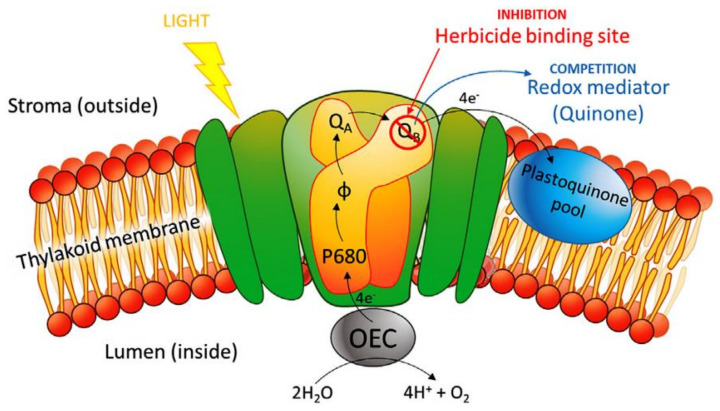
A diagrammatic representation of the photosynthesis inhibition by herbicides. Reproduced from Reference [50] with permission. Copyright 2019, Elsevier.

An amperometric biosensor is a self-contained incorporated device based on the amount of the current ensuing from the oxidation offering exact quantitative analytical information. Generally, these Biosensors have reaction times, energetic ranges, and sensitivities comparable to the Potentiometric biosensors. In amperometric biosensors, the output current of the sensor is analyzed and used for the sensing process. The sensitivity of the amperometric biosensor is determined by comparing the current obtained for the different analyte concentrations. Such biosensors utilized only two electrodes, one for applying the voltage and the other for measuring the current flowing through the device. The amperometric biosensors do not utilize optical or electrochemical devices, but rather depend only on the current measurements. The development of biosensors started from glucose sensing and most of the glucose-sensing involves the catalytic reaction of the enzyme with the glucose oxidase [48,49]. The sensitivity of such glucose sensors is affected by the variation in temperature and pH. In amperometric glucose sensors, cyclic voltametry (CV) and electrochemical impedance spectroscopy (EIS) are the two electrochemical techniques that are used during the sensing process. The sensitivity of an amperometric glucose sensor is measured by the change in current per mM concentration in an area of one-centimeter square, i.e., µA mM^−1^ cm^−2^. The sensitivity of an amperometric glucose sensor ranges from 1 to 15,000 µA mM^−1^ cm^−2^. This is another important parameter while detecting glucose that determines its minimum and maximum concentration to detect the glucose successfully. The range of glucose in the blood (clinical) is between 4.4 and 6.6 mM. Tucci et al. [50] developed an amperometric biosensor for detecting herbicide using Anabaena variabilis cyanobacteria. Diuron and atrazine were used as target herbicides to be detected. The photocurrent produced in the biosensor due to the oxidation of water was found to be dependent upon the concentration of the herbicides. The current starts to decrease with an increase in the concentration of the herbicides. The sensitivity of the biosensor towards atrazine was found to be around 24.6 mA mM^−1^ cm^−2^ with an LOD of 0.56 mM. The mechanism of the inhibition is shown in Figure 6.

The biosensor was selectively used to detect glucose in uric acid, fructose, lactose, ascorbic acid, and mannose. The uric acid in the blood is in the range of 140–430 μM, and any changes in this level lead to kidney stones and gout [51]. The monitoring of uric acid in human blood is very important and biosensors are used effectively for this purpose [52]. Urea is another waste product produced in the kidneys and again variation in its level leads to serious kidney-related diseases [53,54]. The nanorods of ZnO were used to monitor the concentration of urea [54]. An array of ZnO nanotubes was deposited on a silver/silicon substrate to detect cholesterol levels [55]. The sensor showed a sensitivity of about 79.40 μAmM^−1^ cm^−2^ with 0.5 nM LOD. Similarly, ZnO nanorods in a FET transistor, ZnO and multiwalled carbon nanotube (MWCNT) coated Pt/Au electrode, and a composite of CuO and ZnO have been successfully used to detect the cholesterol with good sensitivity and low LOD [56,57,58]. Fang et al. [59] successfully detected the presence of creatine kinase (an enzyme produced during muscle damage of brain or other body muscle) using an electrochemical biosensor made of gold. Cai et al. [60] reported an electrochemical immunosensor with gold and polythionine (PTh-Au) acting as an electrode. The immunosensor had good sensitivity and an LOD of 2.2 pg/mL towards the analytes. Both Fe_3_O_4_ and Ag@Au show good reducing properties towards the hydrogen peroxide. Zhang et al. [61] fabricated an electrochemical biosensor using nanospheres of Fe_3_O_4_ with nanorods of Ag@Au as an electrode. The biosensor demonstrated exceptional and superior behavior towards the reduction of hydrogen peroxide as compared to Fe_3_O_4_ and Ag@Au. Xu et al. [62] developed an amperometric biosensor for the detection of glucose in blood serum. In their work, they self-assembled glucose oxidase and Pt nanoparticles (encapsulated with dendrimers) on a multi-wall carbon nanotube (MWCNT). The biosensor displayed good sensitivity and a low LOD towards glucose. Using a nanocomposite of reduced graphene oxide and gold nanoparticles (rGo/AuNP), Han et al. [63] reported a label-free electrochemical biosensor with enhanced conductivity.

### 3.2. Voltammetric Methods

**Figure 7:**
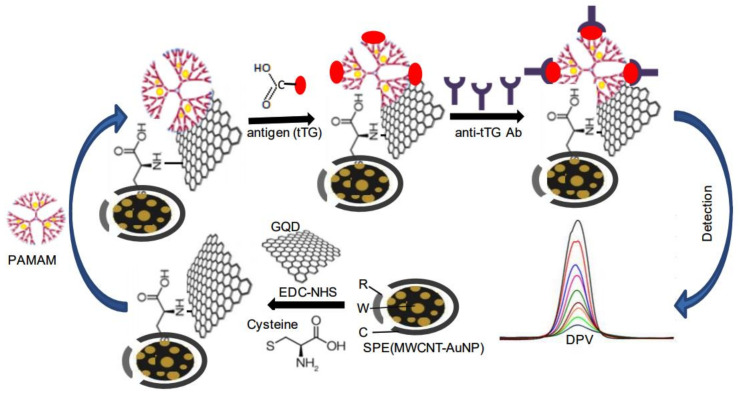
Diagrammatic representation of the biosensor based on DPV and CV to detect anti-tTG in the human celiac disease. Reproduced from Reference [64] with permission. Copyright 2017, Elsevier.

Voltammetry belongs to a category of electro-analytical methods, through which information about an analyte is obtained by varying a potential and then measuring the resulting current. The electrochemical biosensors in which voltammetric techniques such as differential pulse voltammetry (DPV), CV, SWV, and LSV are used for sensing are known as voltammetric biosensors. The voltammetric methods are widely used in sensing platforms due to their low cost, good selectivity, and high sensitivity. Gupta et al. [64] developed a biosensor based on DPV and CV methods to detect anti-iTG antibodies in humans. The biosensor was built by modifying gold nanoparticles with GQD/PAMAM and then embedding them into MWCNT. The biosensors detected successfully the concentration of the antibody as low as 20–50 fgmL^−1^ and are shown diagrammatically in Figure 7 below.

In another published work, Neves et al. [65] fabricated a nano immunosensor working on the CV technique to detect anti-tTG antibodies in the human serum. A carbon electrode that was modified with Au nanoparticles and CNT was taken and onto which the anti-tTG antibodies were printed using screen printing. The sensor showed excellent performance and sensitivity towards the detection of antibodies and can be used for clinical trials. Hianik et al. [66] developed the first aptasensor that worked on voltammetric methods. In this sensor, the DNA aptamers were used to detect thrombin using the DPV technique. The aptamers were deposited on an Au electrode and methylene blue was used as an indicator. The aptasensors can be based on amperometric, potentiometric, voltammetric, conductometric, and impedimetric techniques [67,68]. The voltammetric aptasensors mostly use DPV, CV, and square wave voltammetry (SWV) techniques to detect the antibody. The voltametric biosensor is based on the detection of analyte using the variation in current according to the applied potential. Such biosensors detect both variations in potential and current. The potential is scanned over a wide range and the corresponding result in current and potential are measured. The current produced is directly proportional to the concentration of the target in the electrolyte [69,70]. The voltammetric methods produce low noise and can be used to detect multiple species with different peak potentials in a single scan [71]. The CV technique is used to study the stability, repeatability, and reproducibility of the biosensor [72]. Ly et al. [73] used a CNT electrode modified with bismuth to detect the DNA of Helicobacter pylori present in the gastric tissue of a patient. The biosensor successfully detected the DNA of Helicobacter pylori with a LOD of 6 μg/mL. The response time of the sensor was just 2s and can be used for clinical trials.

### 3.3. Impedimetric Biosensor

**Figure 8:**
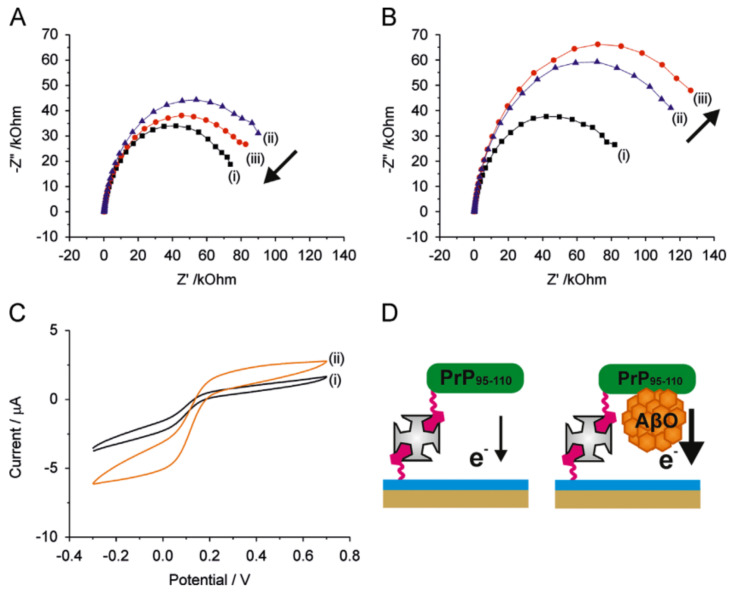
(**A**,**B**) EIS plots. (**C**) CV study of the biosensor towards AβO. (**D**) Diagram showing an increase in the surface conductivity due to AβO. Reproduced from Reference [80] with permission. Copyright 2014, Elsevier.

An impedimetric biosensor is constructed by immobilizing biological recognition elements onto an electrode surface. It reports, through measurement and/or monitoring, the targeted analyte through the output of an electrical impedance signal made proportional to the analyte activity. The electrochemical biosensor in which the variation in impedance is used to detect the analytes or biological entities is called impedance biosensor. The most common technique used in this method is Electrochemical impedance spectroscopy (EIS). Using EIS, the properties of the bulk electrode as well as the processes taking place at the interface of the electrode can be determined easily [74]. The EIS spectrum is obtained as Bode or Nyquist plots, and both are a function of the frequency. The Nyquist plot comprises a semi-circular region on the axis representing the electron-transfer process followed by a straight line depicting the diffusion process. When the transfer of electrons is a quick process, the Nyquist plot only shows a straight line, whereas slow electron transfer is shown by a large semi-circular region [75]. Here, the resistance of the electron transfer is equal to the diameter of the semicircle. On the other hand, Bode’s plot is a type of logarithmic plot where the log of phase(Φ) and impedance (Z) is plotted against frequency (logν). In the EIS, there is a very minute variation in the amplitude of the signal. Moreover, the measurement of impedance does not depend on the presence of redox couples [76], since the biosensor measures a biological event using agents such as antibodies, enzymes, bacteria, viruses, etc. [3,77]. In impedimetric biosensors, the focus is to produce amino, carboxyl, and similar groups on the surface of the electrodes so that the antibodies will be entrapped. This is the most important part while developing an impedimetric biosensor, as it ensures the permanency and repeatability of the sensor. In addition to this, nanomaterials have also been used for such purposes. When the antibody is detected using antigens, the resistance of electron transfer would tend to increase, which eventually decreases the capacitance [4]. The nanoparticles of gold can be used for entrapping antibodies as they do not affect their activity and performance [5]. Canbaz et al. [6] detected HER-3 using the impedimetric technique. Kim et al. [7] used EIS to determine the concentration of bacteria in a fermentor used at the laboratory. The biosensor consists of a PDMS polymer, silicon wafer coated with gold, and a borosilicate glass tube. The EIS was performed for bacteria E. coli for 13 h at a frequency of 0.01 MHz AC. The biosensor successfully detected the presence of E. coli in the fermentor. Seven et al. [78] used EIS to detect MCF-7 cancer cells. On a polypyrrole-NHS electrode, anti-c-cerbB-2 was entrapped using covalent linking. The sensor successfully detected the cancer cells showing the sensitivity of 100–10,000 cells per mL. Ankan et al. [79] investigated the sensing of E. coli bacteria (O157:H7) using an antigen–antibody binding mechanism. The antibody was linked covalently on the surface of PANI film and an EIS study was performed to study the sensitivity and performance of the sensor. The change in impedance upon increasing the concentration of bacteria was measured and recorded. The biosensor was highly sensitive towards E. coli and can be used in labs. Rushworth et al. [80] developed a biosensor for sensing Alzheimer’s amyloid-beta oligomers. By increasing the flow of current through the biosensor, the binding of the oligomer increases, hence decreasing the impedance of the biosensor. The EIS study, CV, and a diagram showing increase in the surface conductivity of the biosensor upon oligomer binding is shown in Figure 8.

In another work, Mao et al. [81] induced SWCNTs into gold electrodes (employing surface modifications) and the electrochemical biosensor was used to detect the DNA sequencing via impedance study. Shirsat et al. [82] reported glucose sensing using multilayer film of polypyrrole and SWCNT on Pt as an electrode. The biosensor displayed excellent sensitivity of 7.06 uA/mM towards glucose.

### 3.4. Potentiometric Biosensors

**Figure 9:**
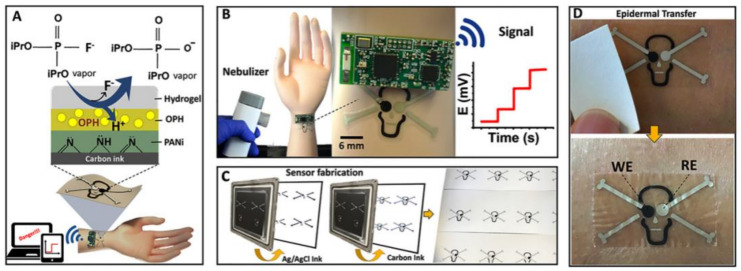
Diagrammatic representation of the tattoo-shaped potentiometric biosensor. (**A**) The concept of the biosensor. (**B**) Designing of the tattoo biosensor. (**C**) The printing process of the biosensor on paper. (**D**) Successful transfer and removal of the tattoo on human skin. Reproduced from Reference [87] with permission. Copyright 2018, Elsevier.

A potentiometric biosensor works on the principle of potential difference between working and reference electrodes. The measured species are not consumed like in the amperometric biosensor. Its response is proportional to the analyte concentration by comparison of its activity to the reference electrode. The great advantage of potentiometric biosensors is their sensitivity and selectivity when a highly stable and accurate reference electrode is used. Potentiometry, a mostly used electrochemical technique in the field of sensors, is cost-effective and used over a wide range of ion concentrations. The sensors developed using potentiometric methods are mostly available in the market. These sensors can be fabricated easily and a reduction in their size does not affect their performance. The use of potentiometric tools in the field of biosensors has opened many new doors for diagnosis and sensing. The two main advantages of using potentiometric biosensors are: the signal produced is in the form of potential and the biochemical component used is the part of the receptor [83]. To monitor the perspiration in humans, potentiometric biosensors in the form of tattoo has been developed [84]. The potentiometric biosensor fabricated by coating polypyrrole on a gold electrode and using mat horseradish peroxidase as a biochemical agent has been developed for detecting tumour, hepatitis B, digoxin, and troponin I [85]. Recently, a potentiometric biosensor with a gold electrode and extended FET transistor was used to detect interleukin with LOD 1 pg mL^−1^ [86]. The acetylcholinesterase was used as an antibody that produced thiol which was adsorbed on the electrode. Mishra et al. [87] fabricated a tattoo shaped biosensor for detecting G-nerve agents using potentiometric methods. In this biosensor, the design of the tattoo sensor was in the form of a skull with one eye working as a reference electrode and the other as a working electrode. The concept, design, printing, and application on human skin are represented diagrammatically in Figure 9.

Canovas et al. [88] developed a method for detecting glucose in human blood serum without dilution. The sample can be tested as received requiring no pre-treatment procedures. A Pt electrode printed on paper was used along with a polyelectrolyte called aquivion. The polyelectrolyte helps in entrapping the glucose enzyme and reducing interference and potential instability. Seema et al. [89] prepared nanoparticles of urease from jack beans and developed a potentiometric biosensor to detect urea in human urine samples. The biosensor displayed an excellent sensitivity of 23 mV/decade and a low LOD of 1 µM/L.

To summarize, various electrochemical methods that have been used for biosensing applications are presented in tabulated form in Table 1.

## 4. Applications of Electrochemical Biosensors

### 4.1. Food Industry

The maintenance of quality and safety of food is one of the major issues of the food industry. Traditionally, spectroscopic and chemical methods have been used to test the safety and quality of food. The traditional methods are laborious, time-consuming, and costly. The biosensors act as an excellent alternative to the traditional methods of food monitoring. The biosensors are efficient, selective, have a fast response time, and are cost-effective [90]. A biosensor made of cobalt phthalocyanine was successfully used to monitor the ageing of beer during storage [91]. E. coli is a pathogen whose presence in the vegetable is an indication of food contamination [92,93]. The electrochemical biosensor (potentiometric) can be used to detect its presence in the vegetables by simply monitoring the pH variation caused by its presence [94]. To detect the presence of organophosphate pesticides in milk, an enzymatic biosensor made of a screen-printed carbon electrode can be used effectively [95]. Artificial sweeteners are used widely in food products and are the reason for various diseases such as diabetes, dental problems, heart diseases, etc. The traditional methods used to detect sweeteners in food products require expertise and a lot of time. The electrochemical biosensors are the new alternatives that can effectively detect artificial sweeteners in food. The signal produced during the electrochemical analysis is analyzed for the presence of artificial sweeteners such as cyclamate and saccharin via MATLAB. The biosensors find huge applications in the food industries where fermentation processes are used. In China, about 90% of the industries use biosensors for monitoring the fermentation processes. Maintaining the quality and safety of the food product during fermentation is extremely important. The biosensors can monitor the condition of the process and the presence of enzymes and biomass in the product. The glutaminase-based electrochemical biosensor chip has proved to be effective in monitoring the fermentation process in various industries and factories. The biosensors can be controlled automatically and are again cost-effective and highly efficient. These can effectively monitor lactate, ethanol, glucose, etc. in the food products during fermentation and act as an indicator for stopping and resuming the fermentation process. The biosensors have gained huge interest in recent years due to their quick and effective monitoring of the fermentation processes [96]. The glucose content in a food product can get altered during the storage process and it gives a direct indication of the quality of the food product [97]. Thus, monitoring the glucose content helps in determining the safety and best-before conditions of a food product. The electrochemical biosensors are the most effective and commonly used biosensors for the monitoring of glucose levels in food products as well as in the human body [98]. The electrochemical biosensors can effectively detect even minute concentrations of harmful metals such as arsenic, lead, cadmium, etc. These have been successfully tested to detect paraoxon, aldicarb, carbaryl, and pesticides in food products [99,100,101,102].

### 4.2. Medical Sciences

The biosensors and their application in detecting the glucose level of diabetic patients are growing very rapidly as they cover 80% of the global biosensors used at home [103,104]. The electrochemical biosensors have been used in the detection of various infectious diseases in the human body such as urinary tract infection, identifying pathogens, microbial bodies, etc. An electrochemical biosensor based on hafnium oxide (HfO_2_) has been developed to detect the infection that occurs after implantation in the human body [105,106]. Cardiovascular diseases and heart failure are the growing diseases in the world leading to the death of millions of people globally [107,108,109,110,111]. The electrochemical biosensors that are effective in detecting such heart failures are also incorporated into digital watches and bands. These watches and bands are efficient and cost-effective and are very useful in saving lives. The electrochemical biosensors based on fluorescence have been used to monitor the level of enzymes in cancer patients. Such biosensors detect the presence of a particular analyte and produce the fluorescent signal which can be detected and measured [112,113]. Such biosensors prove very effective in the earlier detection of the diseases such as inflammation, arthritis, cancer, viral infections, heart-related problems, and metastasis. The electrochemical biosensors are an important part of the drug discovery program. They are used to monitor the working of a drug and are effective in both pre- and post-clinical evaluation [114,115,116,117,118]. Recently, electrochemical biosensors have been successfully used for guiding surgery using imaging techniques and also monitoring the impact of a drug on the disease [119].

### 4.3. Defence

In the current era, biological attacks or warfare is something that we all are aware of and biological warfare agents (BWAs) such as bacteria, virus, toxins, etc. are used in such warfare. The electrochemical biosensors can be effectively used to detect BWAs with high sensitivity and selectivity. The DNA sequencing, monitoring their activity and metabolism, enzymatic action, etc. are some of the principles of such electrochemical biosensor. A potentiometric biosensor was successfully developed to detect botulinum toxin, having an LOD of 10 ng/mL [120]. Gold nanoparticles were combined with magnetic nanoparticles to develop an electrode that can be used to detect the mecA gene, which is a biomarker for methicillin-resistant S. aureus (MRSA). Quantum dots of CdTe were coupled with nanoparticles of silica, and were then used to detect the Epstein-Barr virus via electrochemical methods with an LOD of 1 pg/mL [121]. For detecting Listeria monocytogenes in food samples, a screen-printed carbon electrode was modified using gold nanoparticles. The amperometry result showed an LOD of 2 log CFU/mL [122]. Similarly, *salmonella* spp. [123] and M. tuberculosis [124] have been detected using electrochemical biosensors with high accuracy and sensitivity. The human papillomavirus (HPV), which is related to cervical cancer in humans, has been detected using an electrochemical nucleic acid biosensor [125]. Currently, viruses are being detected using enzyme-linked immunosorbent assay (ELISA) related to viral antigens.

### 4.4. Metabolic Engineering and Plant Biology

The protection of the environment and less dependence on petroleum-driven products are the latest concern on the global scale to cope with global warming. Researchers around the world are searching for products that will be efficient and eco-friendly. Metabolic engineering is one such field where microorganisms are used to produce chemicals, fuels, and pharmaceuticals. Metabolic engineering is an important step towards sustainable development. The biosensors are important in metabolic engineering as they can monitor the metabolism process and help in the controlled production of chemicals and fuels. The imaging and sequencing of DNA have revolutionized the field of plant science. Traditionally, spectroscopic methods were used to monitor the enzymes, receptors, transporters, and substrates. The development of biosensors helps in monitoring such process and is also fast and effective. To monitor and control the level of calcium in live cells, a protein sensor was developed by Roger Tsien’s lab [126,127,128]. FRET biosensors can monitor sucrose and sugar levels during phloem loading-sucrose efflux and the effect of glucose on yeast cells [129,130]. The electrochemical biosensors helped to monitor the effect of pH level on a plant species and play a vital role in genetic engineering [131,132].

## 5. Machine Learning for Biosensors

### 5.1. Improvement in Biosensor by ML

**Figure 10:**
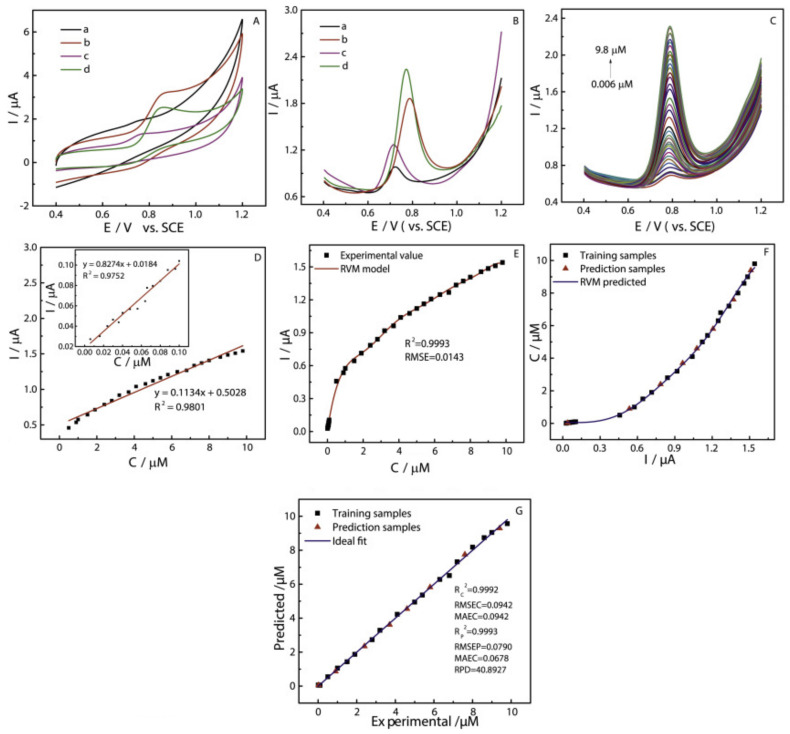
Electrochemical behaviors (**A**) of different modified electrode at the scan rate of 50 mVs−1. (**B**) Corresponding DPV of CBZ at different modified GCE in 0.1 M PBS. (**C**) DPV of CBZ at Ti2C MXene/Au–Ag NS/GCE in 0.1 M PBS. The linear equation of CBZ (**D**) at different concentrations ranging from 0.006 to 9.8 μM. RVM models with concentration as input (**E**) and current as input (**F**) for estimating CBZ concentration obtained by electrochemical. Comparison of the concentration experimental and RVM predicted values of samples (**G**). Reproduced from Reference [132] with permission. Copyright 2021, Elsevier.

Firstly, for specimen or complicated matrices, large sensing data can be efficiently processed by machine learning. Secondly, the gain of ML in biosensors comprises the probability of getting sensible analytical results from disorderly and low-resolution sensing data which could closely overlie on one another. Furthermore, appropriate use of ML methods may find unseen relationships between signals of sensing and parameters of specimen via the visualization of data and interrelations between bioagents and signals. Particularly, raw sensing data can be analyzed by using ML from a biosensor in different ways: Categorization, anomaly detection, noise reduction, and pattern recognition. Based on the target analyte, the algorithms aids to categorize the sensing signals in different manners. It is also observed that the operating conditions inevitably affects the performance of a biosensor. On-site usage of biosensor generally interferes with contamination. In that case, ML plays a very important role in checking the quality of the signal. Because of interferences and biofouling in real samples, the variations in sensor performance can be improved by using ML. It is also observed that sensing signals always contain noise. Hence, it is very important to train to develop the model of ML which extracts the good quality signal from the signal containing noise. Finally, the interpretation of sensing data occurs effectively and easily by developing the patterns and latent objects using ML algorithms [13]. For on-site diagnosis or detection, the ML can be significantly important to aid biosensors that can read out rapidly, accurately, automatically, and directly. Instead of predicting the model for electrochemical biosensor, the optical imaging method assisted by a convolutional neural network (CNN) was also developed to calculate the diagnostic consequences [14]. On the other hand, the pathology workforce takes thirty seconds to interpret the image. Additionally, for designing the desirable biosensors nowadays, ML has been preferred. Metamaterials with negative permittivity and permeability are used to enhance the ability to detect the signals of biosensors based on the surface plasmon resonance (SPR) [15]. To ensure that the resonance is beneficial for SPR biosensors, the process of preparation of metamaterials with different reflectance characteristics is crucial. For predicting the reflectance characteristics of the metamaterial, SPR biosensors like multilayer perceptron (MLP) and Autoencoder (AE) are used. Afterwards, k-means clustering of the metamaterials was introduced for the dimensional reduction with the help of AE and t-StochasticNeighbor embedding (t-SNE). Hence, without experimenting extensively, the designing of the optimized sensing devices can be boosted up with the clustering of the metamaterials. ML plays a crucial role in predicting the mathematical model for the experimental results. Xiaoyu Zhu et al. [132] measured the voltametric behaviors, i.e., differential pulse voltammetry (DPV), using a fabricated electrode at different concentrations of carbendazim (CBZ). The cyclic voltametery (Figure 10A) and DPV (Figure 10B) of different electrodes from the range of 0.4–1.2 V is also calculated. In Figure 10C, it is observed that with increase in the concentration of CBZ, the value of the peak current starts increasing. Meanwhile, a good linear relationship (Figure 10D) between CBZ concentrations and currents (Ipa) is displayed in the range of 0.5–9.8 μM and 0.006–0.1 μM. The Relevance Vector Machine (RVM) model developed with the input of concentrations is represented in Figure 10E. The results obtained from the RVM model of Root mean square error (RMSE) and R-squared were 0.0143 and 0.9993, indicating that the model could be used for detection CBZ in real samples as it shows excellent performance. Figure 10F diagrammatically illustrates the developed RVM model for estimating CBZ concentration using the electrochemical biosensor. The RVM models possess robustness and generalization ability better than the traditional linear regression. Figure 10G depicts the comparisons of the RVM predicted values and experimental values. It is observed that both the predicted concentrations by RVM models and experimental values are in good agreement.

### 5.2. Various Algorithms in ML

In this section, the main aim is to present the procedures of implementation and basic types of advanced algorithms of ML [133]. Conceptually, ML can be defined as a computer program that can gather information from raw data by extracting features. To deal with real-world difficulties, the newly gathered information can become beneficial to make decisions [134]. In the field of biosensors, especially electrochemical, ML is known as a method or tool that can be used for analyzing and processing data for instance concentration of the analytes, extracting features, and for the prediction of the species. It can be classified into unsupervised learning and supervised learning [135]. ML is very important to predict the sensing model for more than one analyte at a particular time. Until today, various algorithms used in ML were known. Those algorithms are preferred, which gives maximum accuracy of the results and give information related to hidden data. When ML algorithms are trained with their target outputs with a group of input data, then it is referred as supervised learning. During the training process, certain predictions can be made by the algorithms on the input data set and the predicted value can be improved by using the given real value, unless the algorithms get the acceptable accuracy. Particularly, in spectrometric biosensors, great progress has been achieved by these to perform regression and categorization. However, in the case of unsupervised learning, labelled training data sets along with their given outputs are not available. The foremost aim is to determine a set of alike examples or to find out the division of data set in the input space (called density estimation). One of the most common unsupervised learning algorithms is k-Means clustering [136].

### 5.3. ML Data Analysis

**Figure 11:**
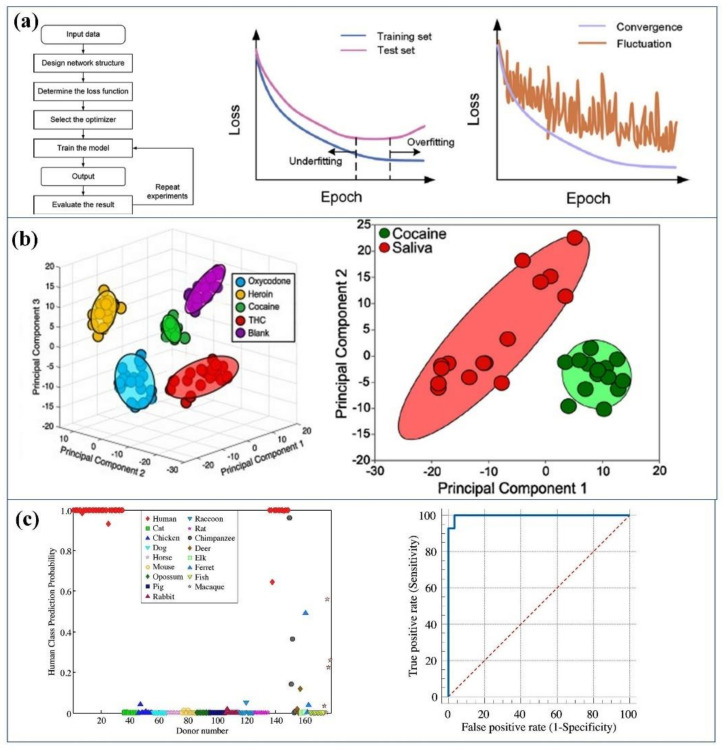
(**a**) Workflow and the scenarios of overfitting and underfitting. Reproduced from Reference [137] with permission. Copyright 2019, Elsevier; (**b**) results of two combined models, i.e., Principal Component Analysis and Support Vector Machine which can be used to distinguish cocaine, oxycodone, tetrahydrocannabinol, and heroin. Reproduced from Reference [139] with permission. Copyright 2018, Elsevier; (**c**) the prediction of partial least squares discriminant analysis (PLS-DA) model for all external human blood donor samples. Reproduced from Reference [140] with permission. Copyright 2018, Elsevier.

The emerging field of biosensor covers both the image data set and sequential data set. The priority in the ML modelling is to develop a suitable model based on the given data sets. After the designing of ML architecture, for a specific biosensor, the workflow will be implemented, which is shown in Figure 11a [137]. The first and foremost requirement is the preprocessing of raw data (sensing data). Various preprocessing methods possess Fourier to transform, denoising, and derivatives. Similarly, the system-specific preprocessing methods must have transformations, normalization, and elimination of baseline drifts and data compression. The overall efficiency of ML model depends on the preprocessing of raw data. For Raman spectroscopy, the requirement of each spectrum is background-subtracted, Savitsky–Golay-smoothed, and [0, 1] min−max scaled [138]. It must be pointed out that the preprocessing of raw data has no guarantee of yielding better results, since it may also remove some informative features from the raw data accidentally.

The preprocessed or raw data set should be split into three subsets, including training set (about 60%), validation set (about 20%), and test set (about 20%). The training data set is used to extract meaningful information and find optimal hyperparameters of the algorithms. The validation data set is applied when tuning hyperparameters. The test data set is employed to report the performance of algorithms. It can also reflect the impact of different hyperparameters [134]. A classic loss curve shows the scenarios of overfitting and underfitting also, and the convergence and fluctuation are determined clearly (Figure 11a). Hyperparameter tuning is a critical task of the sensing data analysis in the validation phase. Parameters for algorithms include the number of hidden neurons, learning rate, batch size, and so forth. To discover the optimal value for each parameter, approaches including grid search, random search, or Bayesian optimization can be applied. Figure 11b shows the combination of two models, i.e., Principal Component Analysis and Support Vector Machine which can be used to distinguish cocaine, oxycodone, tetrahydrocannabinol, and heroin [139]. The developed partial least squares discriminant analysis (PLS-DA) model correctly predicted all external human blood donor samples as human, and 28 of 29 animal blood donor samples as nonhuman (Figure 11c). The ROC curve in Figure 11c had an area under the curve (AUC) of 0.99, indicating that for a randomly chosen sample, the PLS-DA model only had a 1% chance of incorrectly predicting a nonhuman blood sample as being human [140]. A deep learning model was developed based on a SERS data set of exosomes from lung-related cells, and then the model was transferred to predict the lung cancer stage using the SERS data set collected in patient plasma samples [141]. The data set similarity is quantitatively evaluated by the Mahalanobis distance between cancer cell exosomes and plasma exosomes clusters. For 43 cancer patients who are in stages I and II, 90.7% of patients can be accurately predicted using the transferred model. Notably, the similarity of cancer cell exosomes and plasma exosomes has a positive correlation to the stage of cancer. The results demonstrated that the transferred model can predict lung cancer using the SERS of plasma exosomes. The AUC for stage I patients was 0.910, and the AUC for the whole cohort was 0.912.

#### 5.3.1. Support Vector Machine (SVM)

For regression and classification, supervised learning methods used are known as Support vector machines (SVMs) [142]. They are the members of the family of generalized linear classifiers. In tasks like recognition of handwriting, SVM is very popular as it uses pixel maps as input and also gives accuracy that is comparable to sophisticated neural networks with explained characteristics [143]. Many applications are using this in tats like analysis of handwriting, recognition of face, and so on, especially on the application that is based on the classification of pattern and regression. Vapnik [144] developed the Support Vector Machines (SVM) and due to its much better empirical performance, it gained popularity. Conventional neural networks use the principle of Structural Risk Minimization (SRM) that is superior to the traditional Empirical Risk Minimization (ERM) principle [145]. Error in the training data is reduced by the ERM and SRM reduces the upper bound on the risk that is expected. SVMs are nowadays developed to simplify the problems based on the regression as well as problems that are based on the classification [146]. In the detection of waterborne pathogens [147] and cancer diagnosis [148], SVM is used extensively.

#### 5.3.2. Feedforward Artificial Neural Networks (ANN)

**Figure 12:**
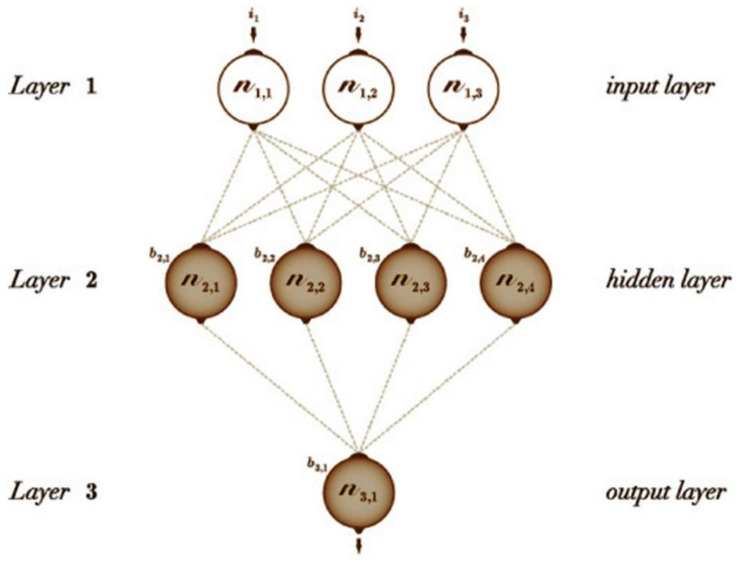
Typical structure of ANN. Reproduced from Reference [149] with permission. Copyright 2018, Elsevier.

The model on simple biological processing of cells (Neurons) and connectivity of the brain is successfully developed through Artificial Neural Network (ANN) as it is a parallel distributed system. Furthermore, a system that is distributed in a parallel way consists of processing elements that can work in parallel and the elements are interconnected (Figure 12) [149]. In addition to this, Artificial Neural Network (ANN) is a system that serves information as distributed system coefficients with a multiple linear regression (MLR) process [150]. Generally, by training the network, information can be gained and need not be known explicitly in advance. Because of that, the adaptability that is combined with the distributed representation in the network is described as the property of recognition of similar patterns as well as the generalisation of abstract patterns within the network input space. Due to this, ANN can degrade and compensate the performance with unreliable inputs and bad training data [151]. Engineers and scientists are provided access to deep learning tools designed by institutions like Microsoft (CNTK), Pytorch, Google (TensorFlow) and universities (Theano). One of the major reasons for the performance of the ANN is significantly affected by the parameters like hidden layer size [152].

#### 5.3.3. Convolutional Neural Network (CNN)

Analyzing images like computed tomography (CT) images, X-ray images, and magnetic resonance images is done by a very efficient deep learning type known as CNN [153,154,155]. Nowadays, solutions based on Artificial Intelligence (AI) are used in various biomedical complications such as detecting breast cancer, brain tumour, etc. [156]. The research communities mostly use this learning method among the different deep learning methods, as convolutional neural networks (CNNs) have provided great accuracy in the classification of the image [157]. On the other hand, there is only one article [158] that explained the categorization of viral and bacterial pneumonia and the applications of deep machine learning algorithms in detecting pneumonia is reported by various research teams [159]. For detecting pneumonia, the parameters of deep layered CNN are varied by the authors in different works. In the radiography of the lungs, interstitial or alveolar patterns of diffuse opacification are observed. Bacterial infection is proof of alveolar infiltration in the radiography of the chest especially in patients with lobar infiltrates [160]. With the enhanced performance in the classification of the images, CNN is very famous. Some important characteristics in an image such as temporal and spatial are extracted by using the convolutional layer that is present in the network. For the reduction in the computation efforts of the computer, the weight-sharing technique is applied [161]. A CNN comprises three building blocks as a max-pooling (subsampling) layer for downsampling the image and reducing the dimensionality and hence reducing the computational efforts of the computer, a fully connected layer for equipping the network with the capability to classify, and a convolution layer for gathering knowledge about characteristics [162].

#### 5.3.4. Recurrent Neural Networks (RNN)

Neural Network in which the output of the previous step is used as an input for the present step is known as Recurrent Neural Network (RNN). In classical neural networks, for the requirement of the prediction of the upcoming word in the sentence, the preceding words are used; therefore, it is important to memorize the preceding words otherwise; inputs and outputs are not dependent on each other. By using a hidden layer, RNN came into being to deal with this issue. A hidden state is the foremost characteristic of RNN, which memorizes some information about the series. In studies related to sequential data, among various learning methods, RNN attracts the researchers significantly [163]. In every recurring round, RNN is compatible with sequential data and time-series as the network structure is particularly developed for representing historical information [164]. Problems based on sequence mapping are widely solved by using RNN such as reinforcement learning, handwriting recognition, speech recognition, and sequence generation because of the characteristic of propagating previous information along with time via recurring connections [165,166,167,168]. For the detection of interactions between proteins and genes, biomedical researchers are now using RNN [169]. According to the latest studies, a promising segmentation of brain tumors can be achieved by training the RNN [170]. An extraordinary type of RNN that is capable of long-term dependencies is Long Short-Term Memory (LSTM) networks [171]. For the detection of modifications in the DNA, Bidirectional RNN with LSTM was designed [172]. The accuracy of the nanopore sequencing has been enhanced with the help of algorithms that are based on RNN [173].

## 6. Challenges and Solution

### 6.1. Challenges

The electrochemical sensors are characterized by three main challenges in their proper functioning, i.e., stability and reproducibility, low LOD value, and real sample sensitivity. These are also the major challenges in the fabrication of electrochemical biosensors.

1. The LOD determines the lowest limit of the analyte that can be detected by a sensor and ideal biosensors must have a very low value of LOD.
2. The reproducibility of sensors is very important when it comes to their fabrication and marketing. The results obtained for a particular sensor must be reproducible to all the similar sensors produced, as testing each sensor will not be possible.
3. Finally, the most important characteristic of a sensor is its application to real samples. If a sensor is not effective in testing a real sample, it cannot be used in the diagnosis. The real samples that are mostly used for electrochemical biosensors are saliva, blood, urine, sweat, body fluid, tears, etc. The real sample collection is itself a challenge; some factors need to be considered for collecting a real sample for detection.
4. The matrix effect in case of electrochemical sensors interferes with the sensor performance. To avoid this matrix effect, the real sample needs to be diluted, but extra dilution may cause deviation from reality. An ideal electrochemical biosensor should sense a real sample without requiring any processing and dilution. Similarly, the samples collected via saliva need dilution before sensing and the pH variation is the problem with the urine samples affecting the peak position. The tear samples due to less complexity have been used for diabetes detection, but the pH variation is again a challenge. Moreover, the concentration of the analytes in the tears produced from irritation and emotion may differ from each other. Moreover, the real samples contain species like protein, fats, etc. that may get adsorbed on the sensor surface and impact the sensitivity and reproducibility of an electrochemical biosensor. The researchers are looking for advanced new materials and techniques (active and passive methods) to address this issue. In the active method, shear forces are produced that prevent the adhesion of the extra species on the sensor surface, whereas in passive methods, polymers are used to make the surface hydrophilic, thus preventing proteins from adsorption. The biosensors developed must be stable under extreme environmental conditions and hence, the stability of the electrochemical biosensors is very important.

### 6.2. Solutions

1. Using nanomaterials might address the stability issue in some cases, but some nanomaterials seem to aggregate and reduce stability.
2. The miniaturization of the electrochemical biosensors and using cheap materials in their fabrication is another step that needs to be taken in making them cheap.
3. Micro-nano fabrication techniques are effective in reducing the size of the electrochemical biosensor. The smaller biosensors would be easy to use and dispose of, can be transported easily, and their application in extreme conditions would involve fewer efforts.
4. The electrochemical biosensors have mostly been confined to the research labs. There needs to be a collaboration between clinics, hospitals, and research labs so that they can be tested in real-life circumstances, which will help in evaluating their performance. Multidisciplinary approach is important for further widespread use and commercialization of biosensors.
5. On a global scale, bacterial diseases are responsible for the greatest number of deaths and illnesses. The electrochemical biosensors can prove effective in sensing these bacterial infections at early stages. These biosensors would also be very useful in detecting new pathogens in the water sources. However, huge efforts on technical and scientific ground will be required to make them more viable. The designing and fabrication process needs to be made more cost-effective. Moreover, the enzymatic electrochemical biosensors are used commonly in the research, but their stability and modification remain a concern. Another challenge is the storage of enzymes.
6. The integration of electrochemical biosensors with POC devices would be a great initiative for application in clinics. Such biosensors would not be affected by the interference species and can detect any concentration of the analyte. In addition to this, nano technology will help in improving the LOD and sensitivity of the electrochemical biosensors.

Since it is a multidisciplinary field, the experts from diverse backgrounds such as materials science, nanotechnology, electronics, chemistry, physics, engineering, medical practitioners, working together will eventually lead to development, enhancement, and eventual commercialization of electrochemical biosensors.

## 7. Future Outlook

**Figure 13:**
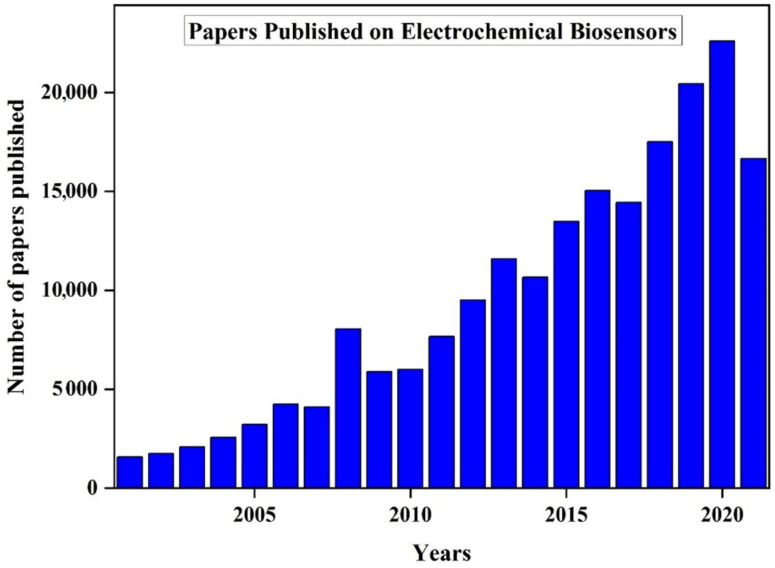
Recent reviews/articles published on electrochemical biosensors from the year 2000 to July 2021. Reproduced from Reference [174]. Copyright 2021, https://www.dimensions.ai/; accessed on 12 August 2021.

The field of electrochemical biosensors has gained immense popularity in the last few decades, and it is quite evident from the publications in this respect, as shown in Figure 13 [174]. As it observed from the figure, the number of publications in “electrochemical biosensors” has increased continuously and a similar trend is expected to be followed in the coming years. The market of the electrochemical biosensors is expected to grow at 9.7% annually and be worth 24 thousand million dollars [119]. In clinical applications, the electrochemical biosensors are used for point-of-care (POC) testing and will reach a value worth of 33 billion dollars.

As mentioned earlier, the electrochemical biosensors are effective in detecting biowarfare agents and this application will become more advanced and available to new species in the future. There is a huge scope for technical and design modifications in this regard. Another field is wearable electrochemical biosensors, and some work has already been done in this field. The wearable biosensors have opened a new market for electrochemical biosensors, and these would prove very effective in sports and athletics. The substrate based on polymers has already been developed. This provides extra flexibility and strength to the wearable biosensor. The wearable biosensors use sweat, saliva, biofluids, etc. for the detection, thus preventing the painful process of taking blood serum for analysis. Moreover, wearable biosensors can be easily installed as bandages, tattoos, contact lenses, cloth, etc. These wearable electrochemical biosensors would be helpful in the food industry, sports, monitoring animal and human health, and defence. Recently, wearable electrochemical biosensors have been developed for monitoring diabetes and this has revolutionized the market. However, the application is in its early stages and would prove to be very effective in monitoring the health of a patient more precisely. The application of nanomaterials in fabricating electrochemical biosensors has enhanced the properties of the biosensors and more such materials need to be searched in the future. The printing of an electrochemical biosensor on the body in the form of a tattoo is another new dimension that has been invented and a lot needs to be done in this regard in the future as this would help in the easy installation of these biosensors. Better biosensing materials and effective bio-fabrication techniques will become important for producing electrochemical biosensors in the future. In addition to this, POC devices that could be easily used at home for monitoring the health of a patient, detecting pathogens in water sources, etc. will be a major concern in the future. To overcome the problems with large-scale production, microelectronics would be a great help in reducing the size of the electrochemical biosensors. In the future, the electrochemical biosensors would accompany the communication technology, and this will help in remotely monitoring the health of a patient. Moreover, the quality and safety of food products can also be managed remotely with such development. The hybrid biosensors, i.e., a combination of two or more biosensors, would help monitor various health aspects. In addition to this, the search for multifunctional materials for biosensing would also provide the same functions. The current global scenario of the COVID-19 pandemic has taken the world by surprise and current testing kits are facing some issues such as time and credibility of the results. The electrochemical biosensors have been successfully used to detect viruses, bacteria, and other antigens. They provide results in less time with efficiency. Thus, electrochemical biosensors would be very helpful in detecting the COVID-19 virus and there is a great scope for developing such biosensors. Recently, degradable electrochemical biosensors have been developed using polyethyleneimine coated over MWCNTs [175]. This paves the way for developing such biosensors without affecting the sustainable development goals. The future has great scope and potential for electrochemical biosensors in various fields of applications. The new and multifunctional biosensing materials need to be searched and the bio-fabrication techniques need to be made more advanced. Microelectronics will help in reducing the size of the biosensors which eventually will be a solution to the various problems.

## 8. Conclusions

Biosensors are analytical devices that convert a biological response into an electrical signal. An ideal biosensor should be temperature and pH-independent, recyclable, and specific. The field of electrochemical biosensors has gained immense popularity in the last few decades, and this is quite evident from the growing number of publications. As observed from the figure, the number of publications in “electrochemical biosensors” has increased continuously and a similar trend is expected to be followed in the coming years. The market of the electrochemical biosensors is expected to grow at 9.7% annually and be worth 24 thousand million dollars. In clinical applications, the electrochemical biosensors are used for point-of-care (POC) testing and will reach a value worth of 33 billion dollars. Biosensors have been applied in many fields namely the food industry, medical field, marine sector, etc., and they provide better stability and sensitivity as compared with the traditional methods. The development and research in electrochemical biosensors are becoming popular in biology, electronics, material science, and engineering. Applications of nanomaterials in biosensors provide opportunities for building up a new generation of biosensor technologies. Nanomaterials improve mechanical, electro-chemical, optical, and magnetic properties of biosensors and are developing towards single-molecule biosensors with high throughput biosensor arrays. The fusion of electrochemical biosensing with nanotechnology and the growing need for inexpensive, mass production of single-use biosensors promises to change the unfortunate fact that glucose test strips are the sole product of the electrochemical biosensor industry to have achieved commercial success. Machine learning helps in interpreting large sensing data and has been used for various applications. The data obtained sometimes are not clear and possess some noise and disturbance. Machine learning helps in analyzing such data and obtain the required results. In the case of biosensors, the presence of impurity affects the performance of the sensor, and machine learning helps in removing signals obtained from the contaminants to obtain a high sensitivity. Presently, electrochemical biosensors are helping in combining biology with electronics. The biosensors are becoming efficient, smaller, and cost-effective. In the future, the electrochemical biosensor will revolutionize the field of diagnosis, health care, food security, and defense.

## Figures and Tables

**Table 1:** List of electrochemical biosensors along with their applications.

Method	Target	Biological Element	Target Matrix	Transducer Element	Ref.
Amperometric	Cholesterol	Cholesterol oxidase	Human serum	Prussian Blue modified SPE	[16]
Amperometric	Lactate	Lactate oxidase	Wine	Prussian Blue modified SPE	[17]
Amperometric	Polyamines	Polyamine oxidase, spermine oxidase	Food	Prussian Blue modified SPE	[18]
Amperometric	Lysine	Lysine oxidase	Cheese	Pt electrode	[19]
Amperometric	Glucose	Glucose oxidase	Transdermal fluid	Transdermal microneedles	[20]
Amperometric	Glucose	Glucose oxidase	-	Gold nanoelectrode	[21]
Amperometric	Ethanol	Alcohol dehydrogenase	wine	Polyaniline doped modified SPE	[22]
Amperometric	Antioxidant capacity	Superoxide disumlase	Fruit juice and berries	Pt electrode	[23]
Amperometric differential	Antioxidant capacity+ ascorbate	Ascorbate oxidase	Fruit juice	Fullerene modified graphite	[24]
Amperometric inhibition	Atrazine	Tyrosinase	Drinking water	Carbon modified SPE	[25]
Amperometric	Oxygen profile	Biliribine oxidase	Microbial fuel cell	Pt electrode	[26]
Label-free evanescent wave	IgG	Antibody	Human serum	Titania–silica-coated long period gratings optical fibers	[28]
Label-free CCD + software for imaging	Prostate specific antigen	Antibody	Human serum	Dense arrays of micropillars	[29]
Label-free field effect transistor	Interleukin 4	Antibody	Human serum	Organic transistor	[30]
Voltametric/ impedimetric	Aflatoxin B1	Aptamer	Peanuts and peanuts corn snacks	Dendrimer- modified gold electrode	[32]
Label-free, piezoelectric using 2 different aptamers	Metalloproteinase 9	Aptamers	Human serum	Quartz crystal microbalance	[33]
Colorimetric, aggregation using 2 aptamers	DNA methylation	Aptamers for α-thrombin	DNA	Au coated magnetic nanoparticles	[34]
Impedimetric	Human epidermal growth factor receptor 2	Antibody	Human serum	Au–nano-particles on SPE	[35]
